# Supplementary material for: A Likelihood Approach for Real-Time Calibration of Stochastic Compartmental Epidemic Models
Source: PLoS Comput Biol. 2017 Jan 17;13(1):e1005257. doi: 10.1371/journal.pcbi.1005257 (PMC5240920; doi:10.1371/journal.pcbi.1005257)
Supplement: S1 File — (TAR.GZ) [file pcbi.1005257.s014.tar.gz › HSPH_Online-SI-Revision/MSS10/n10-severe/n10-severe_NEW2_table-8.pdf]

1	newly 3 weeks spec	291.	495.95	{164., 793.}	87.4192	0.287176	newly 3 weeks cumul	664.	1080.52	{341., 1737.}	79.6566	0.266972	newly 5 weeks spec	473.	567.91	{256., 929.}	39.833	0.189925	newly 5 weeks cumul	1512.	2224.88	{973., 3383.}	62.9802	0.227739	newly 59 weeks cumul	6569.	4174.22	{3010., 5634.}	39.8727	0.248086
2	newly 3 weeks spec	216.	163.8	{6., 309.}	55.0556	0.527861	newly 3 weeks cumul	425.	406.17	{53., 835.}	53.0988	0.334797	newly 5 weeks spec	426.	187.53	{0., 417.}	63.0446	0.881724	newly 5 weeks cumul	1146.	779.28	{69., 1535.}	52.8272	0.443192	newly 61 weeks cumul	6678.	1904.51	{69., 3699.}	73.2598	0.852077
3	newly 3 weeks spec	232.	245.85	{66., 432.}	53.056	0.255193	newly 3 weeks cumul	460.	526.38	{154., 874.}	52.9739	0.232185	newly 5 weeks spec	491.	373.5	{97., 648.}	41.3727	0.308573	newly 5 weeks cumul	1296.	1221.23	{340., 2032.}	44.2199	0.245847	newly 63 weeks cumul	5504.	3938.06	{2753., 5282.}	33.9095	0.204355
4	newly 3 weeks spec	222.	230.26	{19., 518.}	70.5495	0.494474	newly 3 weeks cumul	397.	453.88	{49., 995.}	68.6751	0.367374	newly 5 weeks spec	435.	406.04	{0., 934.}	63.6092	0.64281	newly 5 weeks cumul	1174.	1190.9	{64., 2742.}	65.8501	0.426679	newly 58 weeks cumul	6313.	3746.36	{187., 5641.}	41.2831	0.42257
5	newly 3 weeks spec	228.	139.73	{14., 254.}	55.5658	0.50574	newly 3 weeks cumul	503.	336.74	{70., 574.}	46.004	0.339961	newly 5 weeks spec	460.	178.74	{0., 361.}	64.3261	0.876508	newly 5 weeks cumul	1330.	675.56	{71., 1225.}	55.5534	0.478117	newly 58 weeks cumul	6047.	2126.9	{71., 3974.}	64.8272	0.711287
6	newly 3 weeks spec	208.	158.78	{93., 198.}	42.0962	0.210307	newly 3 weeks cumul	401.	316.44	{212., 373.}	37.0374	0.181	newly 5 weeks spec	328.	367.04	{234., 418.}	29.1098	0.0982135	newly 5 weeks cumul	983.	936.25	{603., 1048.}	30.53	0.1241	newly 59 weeks cumul	5568.	8270.67	{7971., 8536.}	48.7505	0.171401
7	newly 3 weeks spec	360.	452.86	{346., 551.}	31.6333	0.122555	newly 3 weeks cumul	893.	1054.56	{800., 1274.}	24.4188	0.0982224	newly 5 weeks spec	397.	593.59	{434., 715.}	54.1436	0.188476	newly 5 weeks cumul	1675.	2182.57	{1637., 2612.}	35.1003	0.1323	newly 58 weeks cumul	4740.	5842.22	{3509., 6993.}	36.6979	0.145716
8	newly 3 weeks spec	247.	382.9	{285., 497.}	57.1498	0.188713	newly 3 weeks cumul	499.	713.83	{521., 906.}	45.6493	0.157478	newly 5 weeks spec	477.	842.4	{660., 1017.}	77.065	0.242192	newly 5 weeks cumul	1346.	2163.35	{1677., 2722.}	61.6085	0.201685	newly 62 weeks cumul	5812.	8374.85	{7626., 8989.}	44.0958	0.157669
9	newly 3 weeks spec	71.	264.1	{31., 529.}	296.592	0.610554	newly 3 weeks cumul	127.	450.61	{63., 836.}	274.433	0.526937	newly 5 weeks spec	311.	682.16	{96., 1337.}	154.051	0.555903	newly 5 weeks cumul	564.	1609.64	{196., 3214.}	212.571	0.529724	newly 66 weeks cumul	6105.	6178.07	{3144., 9061.}	35.3597	0.285224
10	newly 3 weeks spec	214.	454.99	{87., 830.}	135.098	0.428005	newly 3 weeks cumul	335.	725.76	{168., 1285.}	136.263	0.368232	newly 5 weeks spec	555.	1088.99	{231., 1759.}	116.849	0.481105	newly 5 weeks cumul	1279.	2624.75	{668., 4571.}	125.671	0.394942	newly 66 weeks cumul	6231.	6684.19	{3559., 9105.}	30.6569	0.220574
11	newly 3 weeks spec	107.	818.05	{93., 1639.}	678.383	0.806603	newly 3 weeks cumul	156.	1802.3	{206., 4055.}	1064.37	0.895994	newly 5 weeks spec	375.	432.43	{125., 695.}	57.8693	0.369105	newly 5 weeks cumul	750.	2866.37	{474., 5052.}	298.844	0.586579	newly 65 weeks cumul	5214.	3921.04	{1605., 5179.}	25.7434	0.234614
12	newly 3 weeks spec	89.	177.27	{63., 301.}	112.191	0.318674	newly 3 weeks cumul	220.	300.52	{117., 489.}	57.8364	0.210554	newly 5 weeks spec	266.	606.75	{191., 1021.}	140.793	0.381796	newly 5 weeks cumul	648.	1259.75	{460., 2105.}	108.162	0.313291	newly 65 weeks cumul	5759.	8080.44	{5870., 9171.}	46.5376	0.190154
13	newly 3 weeks spec	161.	92.65	{26., 186.}	60.9752	0.514426	newly 3 weeks cumul	272.	181.66	{59., 362.}	53.6544	0.367867	newly 5 weeks spec	444.	227.05	{40., 497.}	66.0023	0.67131	newly 5 weeks cumul	1004.	559.72	{160., 1329.}	62.3108	0.493834	newly 62 weeks cumul	6792.	6168.25	{1763., 8437.}	26.7761	0.281621
14	newly 3 weeks spec	177.	186.01	{44., 311.}	47.4068	0.299273	newly 3 weeks cumul	299.	345.15	{106., 568.}	48.786	0.230305	newly 5 weeks spec	540.	497.95	{62., 882.}	42.3574	0.378513	newly 5 weeks cumul	1231.	1168.61	{225., 1975.}	41.8367	0.273358	newly 64 weeks cumul	5655.	7013.15	{3006., 9229.}	46.4271	0.282835
15	newly 3 weeks spec	125.	1259.76	{0., 3730.}	963.872	1.16209	newly 3 weeks cumul	202.	1946.73	{11., 6383.}	912.876	0.881691	newly 5 weeks spec	451.	898.99	{0., 2364.}	165.843	0.981869	newly 5 weeks cumul	842.	4008.57	{17., 8811.}	430.191	0.917862	newly 68 weeks cumul	6627.	5899.09	{17., 9385.}	44.3255	0.618184
16	newly 3 weeks spec	329.	965.43	{271., 1845.}	204.125	0.436782	newly 3 weeks cumul	674.	1834.07	{550., 3617.}	183.853	0.396382	newly 5 weeks spec	678.	773.9	{275., 1349.}	42.1386	0.216484	newly 5 weeks cumul	1835.	3584.53	{1650., 5912.}	103.937	0.30801	newly 64 weeks cumul	6828.	6158.95	{3586., 8854.}	25.844	0.167097
17	newly 3 weeks spec	187.	302.11	{155., 464.}	73.1604	0.251231	newly 3 weeks cumul	387.	554.52	{296., 887.}	55.1886	0.190489	newly 5 weeks spec	326.	740.19	{374., 1061.}	134.77	0.390196	newly 5 weeks cumul	968.	1802.17	{1008., 2720.}	94.499	0.2898	newly 64 weeks cumul	6543.	7251.75	{3991., 9255.}	28.621	0.1396
18	newly 3 weeks spec	201.	416.71	{319., 501.}	109.318	0.344531	newly 3 weeks cumul	460.	820.44	{661., 979.}	80.1217	0.257104	newly 5 weeks spec	415.	781.31	{594., 913.}	89.8627	0.277037	newly 5 weeks cumul	1164.	2204.3	{1703., 2586.}	91.0825	0.281808	newly 62 weeks cumul	6707.	8109.83	{6922., 9122.}	25.0769	0.0991581
19	newly 3 weeks spec	266.	144.6	{115., 179.}	45.6391	0.269499	newly 3 weeks cumul	588.	346.38	{287., 401.}	41.0918	0.233204	newly 5 weeks spec	499.	218.85	{177., 265.}	56.1423	0.36382	newly 5 weeks cumul	1512.	741.13	{629., 866.}	50.9835	0.313287	newly 58 weeks cumul	6578.	6924.21	{6738., 7125.}	5.73563	0.0242492
20	newly 3 weeks spec	163.	158.07	{23., 282.}	55.	0.453605	newly 3 weeks cumul	399.	407.23	{63., 653.}	50.0977	0.302263	newly 5 weeks spec	300.	194.35	{0., 423.}	56.45	0.686356	newly 5 weeks cumul	931.	780.61	{78., 1389.}	47.0602	0.356883	newly 58 weeks cumul	5881.	1830.41	{78., 3624.}	68.8759	0.757467
21	newly 3 weeks spec	178.	889.86	{431., 1319.}	403.753	0.679129	newly 3 weeks cumul	310.	1348.51	{661., 1998.}	338.61	0.61463	newly 5 weeks spec	488.	1723.34	{1235., 1990.}	255.488	0.55076	newly 5 weeks cumul	1116.	4594.39	{3066., 5752.}	314.169	0.609509	newly 66 weeks cumul	6367.	8588.21	{7087., 9354.}	35.9493	0.131278
22	newly 3 weeks spec	136.	710.45	{324., 1460.}	422.39	0.651038	newly 3 weeks cumul	315.	1421.73	{658., 3226.}	351.698	0.575225	newly 5 weeks spec	337.	857.52	{391., 1186.}	159.769	0.39921	newly 5 weeks cumul	870.	3085.84	{1849., 4611.}	254.694	0.528307	newly 64 weeks cumul	5004.	7782.81	{4763., 8981.}	57.4363	0.186971
23	newly 3 weeks spec	247.	286.28	{140., 406.}	36.5506	0.161468	newly 3 weeks cumul	543.	552.73	{273., 746.}	25.7772	0.130773	newly 5 weeks spec	429.	612.24	{282., 855.}	55.4452	0.201599	newly 5 weeks cumul	1291.	1609.74	{811., 2210.}	40.8056	0.16557	newly 63 weeks cumul	5813.	7160.11	{4091., 9099.}	35.7063	0.141094
24	newly 3 weeks spec	98.	308.7	{22., 1178.}	263.878	0.470262	newly 3 weeks cumul	155.	524.54	{61., 1827.}	265.523	0.364719	newly 5 weeks spec	358.	341.77	{36., 971.}	77.9916	0.52977	newly 5 weeks cumul	700.	1239.86	{143., 4412.}	138.397	0.42107	newly 64 weeks cumul	6876.	6866.94	{4847., 8764.}	24.9401	0.225379
25	newly 3 weeks spec	16.	493.16	{46., 1064.}	2987.13	1.28429	newly 3 weeks cumul	53.	721.01	{88., 1522.}	1263.98	0.951701	newly 5 weeks spec	207.	1288.44	{211., 2070.}	534.242	0.797489	newly 5 weeks cumul	318.	2973.97	{409., 6006.}	843.67	0.880357	newly 67 weeks cumul	5792.	7475.74	{5232., 9326.}	40.0846	0.207466
26	newly 3 weeks spec	172.	627.53	{258., 967.}	268.134	0.552369	newly 3 weeks cumul	282.	921.48	{404., 1429.}	230.014	0.486639	newly 5 weeks spec	397.	1655.11	{1077., 1971.}	318.904	0.641223	newly 5 weeks cumul	956.	3826.92	{2106., 5129.}	302.621	0.594368	newly 66 weeks cumul	5226.	8888.48	{7943., 9352.}	72.0708	0.253916
27	newly 3 weeks spec	68.	58.97	{2., 119.}	105.221	0.746103	newly 3 weeks cumul	149.	98.63	{8., 181.}	91.	0.675228	newly 5 weeks spec	292.	188.26	{1., 436.}	94.1781	1.05173	newly 5 weeks cumul	586.	398.03	{14., 803.}	94.7423	0.796083	newly 67 weeks cumul	6353.	6380.18	{52., 9186.}	40.0283	0.426074
28	newly 3 weeks spec	54.	72.34	{10., 160.}	81.1111	0.381286	newly 3 weeks cumul	104.	124.45	{22., 262.}	68.3173	0.311502	newly 5 weeks spec	285.	280.07	{28., 637.}	68.7474	0.458015	newly 5 weeks cumul	567.	556.98	{63., 1289.}	65.7637	0.393073	newly 66 weeks cumul	5472.	7730.19	{5001., 9272.}	51.8101	0.260494
29	newly 3 weeks spec	78.	45.41	{0., 100.}	100.244	1.13061	newly 3 weeks cumul	105.	63.21	{2., 134.}	96.2762	0.871653	newly 5 weeks spec	382.	237.37	{0., 747.}	102.825	1.36375	newly 5 weeks cumul	647.	421.87	{3., 1148.}	104.447	1.10956	newly 68 weeks cumul	5977.	6286.61	{4., 9320.}	51.1292	0.794531
30	newly 3 weeks spec	42.	22.98	{0., 27.}	115.143	1.36746	newly 3 weeks cumul	59.	31.98	{1., 39.}	105.492	0.953933	newly 5 weeks spec	274.	106.56	{0., 149.}	108.825	1.83099	newly 5 weeks cumul	436.	207.75	{2., 255.}	113.709	1.38411	newly 68 weeks cumul	6478.	5533.42	{2., 9202.}	50.1127	0.988705
31	newly 3 weeks spec	233.	1298.01	{516., 1773.}	461.052	0.74225	newly 3 weeks cumul	480.	2601.16	{958., 4015.}	446.154	0.701955	newly 5 weeks spec	432.	672.89	{223., 1241.}	80.9051	0.291429	newly 5 weeks cumul	1241.	4357.14	{2907., 5168.}	254.525	0.557933	newly 65 weeks cumul	5273.	5644.49	{4693., 8973.}	19.2583	0.0964927
32	newly 3 weeks spec	278.	210.43	{54., 415.}	45.9748	0.391147	newly 3 weeks cumul	694.	610.98	{174., 1063.}	41.1671	0.278735	newly 5 weeks spec	361.	173.81	{14., 406.}	57.6593	0.658764	newly 5 weeks cumul	1404.	983.71	{271., 1853.}	43.609	0.342351	newly 58 weeks cumul	4849.	1709.91	{334., 3350.}	64.7369	0.62941
33	newly 3 weeks spec	255.	361.23	{235., 507.}	50.7725	0.188253	newly 3 weeks cumul	589.	793.98	{551., 1075.}	42.7267	0.15691	newly 5 weeks spec	409.	530.39	{386., 666.}	38.2763	0.162571	newly 5 weeks cumul	1337.	1778.01	{1298., 2392.}	40.8145	0.158397	newly 58 weeks cumul	5959.	4695.82	{3445., 5364.}	21.1979	0.12425
34	newly 3 weeks spec	330.	336.63	{212., 451.}	22.8939	0.108969	newly 3 weeks cumul	818.	795.57	{559., 1023.}	19.324	0.0939154	newly 5 weeks spec	455.	427.08	{296., 543.}	17.578	0.0930572	newly 5 weeks cumul	1672.	1620.15	{1110., 2057.}	17.4133	0.0873173	newly 58 weeks cumul	6027.	4213.17	{2999., 4910.}	30.842	0.172311
35	newly 3 weeks spec	124.	234.3	{45., 464.}	116.145	0.445471	newly 3 weeks cumul	254.	406.19	{101., 753.}	89.4449	0.340669	newly 5 weeks spec	365.	634.38	{107., 1218.}	103.277	0.506404	newly 5 weeks cumul	843.	1461.49	{284., 2738.}	101.145	0.40094	newly 65 weeks cumul	5774.	6712.53	{1924., 9224.}	45.0324	0.343693
36	newly 3 weeks spec	206.	216.98	{19., 405.}	60.2427	0.465517	newly 3 weeks cumul	457.	492.94	{67., 922.}	58.3107	0.34044	newly 5 weeks spec	363.	291.89	{11., 546.}	54.0358	0.612164	newly 5 weeks cumul	1101.	1046.34	{117., 1990.}	52.6067	0.374255	newly 61 weeks cumul	6168.	2942.85	{134., 4109.}	58.5109	0.588704
37	newly 3 weeks spec	223.	312.3	{139., 467.}	57.0224	0.180201	newly 3 weeks cumul	420.	545.17	{283., 789.}	47.5405	0.157166	newly 5 weeks spec	351.	760.17	{444., 1110.}	120.288	0.326197	newly 5 weeks cumul	1055.	1825.02	{1018., 2665.}	78.7393	0.231417	newly 64 weeks cumul	5466.	8455.97	{7617., 9264.}	57.0397	0.198431
38	newly 3 weeks spec	171.	236.75	{27., 559.}	94.5322	0.493015	newly 3 weeks cumul	342.	496.78	{72., 991.}	87.1813	0.35197	newly 5 weeks spec	402.	335.62	{29., 758.}	67.99	0.631095	newly 5 weeks cumul	991.	1125.41	{171., 2637.}	76.106	0.4158	newly 64 weeks cumul	6742.	2966.04	{335., 5410.}	62.1501	0.635025
39	newly 3 weeks spec	270.	369.42	{236., 482.}	43.0296	0.152652	newly 3 weeks cumul	603.	802.9	{528., 1019.}	39.7313	0.143545	newly 5 weeks spec	373.	566.52	{390., 715.}	54.5469	0.184202	newly 5 weeks cumul	1298.	1845.75	{1216., 2348.}	46.5092	0.16259	newly 60 weeks cumul	6183.	5525.93	{4726., 7750.}	18.4585	0.0852947
40	newly 3 weeks spec	213.	225.24	{38., 399.}	55.7653	0.310131	newly 3 weeks cumul	503.	513.12	{132., 953.}	49.0497	0.249113	newly 5 weeks spec	419.	317.49	{57., 572.}	42.8425	0.344457	newly 5 weeks cumul	1236.	1110.42	{250., 1902.}	44.2184	0.266101	newly 60 weeks cumul	6409.	3221.2	{1324., 4041.}	49.9379	0.360883
41	newly 3 weeks spec	69.	162.7	{1., 257.}	241.043	0.859272	newly 3 weeks cumul	109.	222.96	{5., 389.}	205.284	0.68311	newly 5 weeks spec	305.	451.84	{1., 1589.}	155.889	1.03935	newly 5 weeks cumul	561.	985.46	{8., 2738.}	181.526	0.855767	newly 68 weeks cumul	6138.	7079.52	{3302., 9323.}	42.1909	0.382724
42	newly 3 weeks spec	203.	35.61	{15., 60.}	82.4581	0.846402	newly 3 weeks cumul	358.	78.61	{43., 126.}	78.0419	0.71366	newly 5 weeks spec	454.	70.23	{22., 119.}	84.9934	0.930515	newly 5 weeks cumul	1061.	199.6	{89., 308.}	81.1876	0.803433	newly 59 weeks cumul	6484.	6330.04	{4508., 7793.}	15.5543	0.120115
43	newly 3 weeks spec	127.	86.81	{2., 153.}	97.7244	0.846079	newly 3 weeks cumul	210.	133.22	{9., 232.}	88.7238	0.671783	newly 5 weeks spec	451.	268.05	{1., 863.}	85.102	0.973959	newly 5 weeks cumul	888.	569.77	{17., 1572.}	88.7736	0.789361	newly 67 weeks cumul	6305.	7036.47	{1131., 9261.}	39.9326	0.409566
44	newly 3 weeks spec	233.	189.69	{49., 336.}	40.8712	0.300395	newly 3 weeks cumul	482.	402.32	{166., 703.}	35.9046	0.227038	newly 5 weeks spec	403.	361.79	{72., 639.}	41.8635	0.364495	newly 5 weeks cumul	1184.	1035.74	{319., 1868.}	38.6233	0.254314	newly 61 weeks cumul	5552.	5535.94	{992., 8270.}	39.4186	0.299511
45	newly 3 weeks spec	215.	264.86	{128., 413.}	47.1535	0.187618	newly 3 weeks cumul	394.	457.97	{231., 703.}	40.5051	0.167509	newly 5 weeks spec	444.	775.25	{374., 1121.}	83.9932	0.263741	newly 5 weeks cumul	1183.	1727.81	{819., 2578.}	59.4193	0.206627	newly 64 weeks cumul	6681.	8362.72	{7625., 9316.}	28.3012	0.109838
46	newly 3 weeks spec	191.	313.98	{49., 644.}	103.927	0.410484	newly 3 weeks cumul	452.	631.9	{119., 1181.}	77.5398	0.321613	newly 5 weeks spec	337.	495.77	{53., 985.}	87.2671	0.482623	newly 5 weeks cumul	1052.	1552.53	{259., 3031.}	85.7443	0.361885	newly 63 weeks cumul	5089.	3826.52	{1341., 5643.}	30.1949	0.269737
47	newly 3 weeks spec	210.	224.75	{61., 417.}	56.3286	0.306364	newly 3 weeks cumul	472.	486.16	{163., 824.}	47.661	0.258808	newly 5 weeks spec	303.	386.77	{77., 672.}	67.033	0.346257	newly 5 weeks cumul	1066.	1188.45	{305., 2020.}	53.5863	0.276628	newly 64 weeks cumul	5261.	3671.32	{1799., 5258.}	32.4611	0.239292
48	newly 3 weeks spec	153.	151.11	{59., 233.}	36.8039	0.189221	newly 3 weeks cumul	281.	306.21	{145., 472.}	36.5587	0.16374	newly 5 weeks spec	442.	344.59	{161., 524.}	35.8801	0.243652	newly 5 weeks cumul	972.	886.15	{403., 1372.}	33.6656	0.184445	newly 61 weeks cumul	6845.	6184.26	{3262., 8419.}	26.2457	0.170669
49	newly 3 weeks spec	191.	329.81	{43., 667.}	111.607	0.428956	newly 3 weeks cumul	421.	678.97	{117., 1233.}	99.2993	0.345597	newly 5 weeks spec	403.	449.45	{66., 706.}	53.3275	0.369038	newly 5 weeks cumul	1126.	1550.41	{189., 2990.}	76.0115	0.327902	newly 63 weeks cumul	5314.	3921.47	{1506., 8295.}	41.3062	0.306984
50	newly 3 weeks spec	165.	421.19	{3., 1212.}	237.242	0.814697	newly 3 weeks cumul	248.	611.48	{20., 1548.}	222.879	0.582176	newly 5 weeks spec	521.	867.1	{0., 2253.}	138.649	0.944654	newly 5 weeks cumul	1082.	2148.69	{25., 6538.}	170.578	0.697792	newly 68 weeks cumul	6795.	6480.23	{25., 9262.}	38.0661	0.511374
